# Evaluation of tree lucerne (*Chamaecytisus palmensis*) dried leaves as a substitution for concentrate mixture on biological performance and socioeconomic of Washera sheep fed on desho grass hay

**DOI:** 10.1002/vms3.376

**Published:** 2020-10-10

**Authors:** Shewaye Hailecherkos, Bimrew Asmare, Yeshambel Mekuriaw

**Affiliations:** ^1^ School of Animal Science and Veterinary Medicine Bahir Dar University Bahir Dar Ethiopia

**Keywords:** daily weight gain, digestibility, fodder shrub, ovine, tree lucerne

## Abstract

**Background:**

The experiment was conducted to evaluate the effect of tree lucerne dried leaves (TLDL) as a substituting supplement for noug seed cake, and wheat bran concentrate mixtures (CM) on feed intake, digestibility, growth, carcass characteristics and economic benefits using Washera sheep fed desho grass hay as a basal diet.

**Methods:**

Twenty‐five male Washera sheep with an initial body weight of 16 ± 4.01 kg (mean ± *SD*) were used for both growth and digestibility trials which lasted for 90 and 10 days, respectively The experiment was conducted using a randomized complete block design having five blocks with five treatments (T1 = 0% TLDL + 100% CM; T2 = 25% TLDL + 75% CM; T3 = 50% TLDL + 50% CM; T4 = 75% TLDL + 25% CM and T5 = 100% TLDL + 0% CM).

**Results:**

Significant (*p* < .05) differences were observed among treatments in total dry matter (DM), nutrients and metabolizable energy (ME) intake; as the level of TLDL increases total DM and nutrient intake decreased (*p* < .05) except neutral detergent fibre intake (NDFI) and acid detergent fibre intake (ADFI). Digestibility of DM, organic matter (OM) and crude protein (CP) were significantly reduced (*p* < .001) among the treatments increasing the substitution level of TLDL for local concentrate mixture, but non‐significant for fibre fractions digestibility. Similarly, body weight, average daily gain and feed conversion efficiency were significantly different (*p* < .01) and highest in T1 than other treatments, whereas lowest values of the same parameters were observed for T5. Non‐significant difference among treatments was observed in almost all of the carcass characteristics except for rib‐eye area that showed significant difference among treatment and was highest in T1 and T2 (low level of TLDL inclusion). The economic analysis showed that supplementation TLDL was economically feasible in which the net return of the treatments were 31.66 (T1), 30.68 (T2) and 30.34 (T3), 27.88 (T4) and 26.32 (T5) USD (United States Dollar).

**Conclusions:**

It was concluded that TLDL could be used as alternative feed source by replacing concentrate mixture up to 75% (T4) which enhanced nutrient intake, digestibility and growth performance of Washera sheep. Finally, TLDL supplementation is recommended as replacement of CM up to 75% for its biological and economic feasibility.

## Graphical abstract

The experiment was conducted to evaluate the effect of tree lucerne dried leaves and to determine the optimum level of substituting for concentrate mixtures (CM) on feed intake, nutrient digestibility, body weight change, carcass characteristics and economic benefits of using Washera sheep fed on desho grass hay as a basal diet.

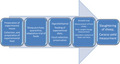

**Abbreviations**
CP: crude protein
FCE: feed conversion efficiency
ME: metabolizable energy
NI: net income
NSC: noug seed cake
TEOC: total edible offal component
TLDL: tree lucerne dried leaves
TNEOC: total none edible offal component
TR: total return
TVC: total variable cost
WB: wheat bran

## 1 INTRODUCTION

The recent population of cattle, sheep and goats in Ethiopia are estimated to be about 59.5, 30.7 and 30.2 million, respectively, reported in the country (CSA [Central Statistical Agency], 2017). Among these livestock species in the country, sheep is the second dominant in terms of population next to cattle (CSA, 2017; Mekuriaw and Asmare, 2018). In Ethiopia, despite large number of sheep, productivity is very low which mainly is caused by many factors like feed deficit, low genetic potential of sheep and prevalence of disease. But shortage of feed in terms of quality and quantity is the critical one (Kebede, Assefa, Feyissa, & Mengistu, 2015). The major feed resources in the country are natural pasture and crop residues (CSA, 2017) which are critically deficit in important nutrients like protein and minerals and could not satisfy the maintenance requirement of ruminant animals and reflected in weight loss if fed alone without supplement (Tefera, Getachew, & Mengistu, 2015; Tekliye et al., 2018). So, supplementation with multipurpose fodder tree/shrub leaves and foliage is globally as well as locally recommended (Asmare & Mekuriaw, 2019; Bakshi & Wadhwa, 2007; Balehegn, 2017). As far these multipurpose fodder species integrated in the farming system and used as ruminant livestock feed, they could be sustainably available to the farmers for livestock industry. Among several browse species in the tropics, tree lucerne (*Chamaecytisus palmensis*) is well adapted and evergreen, hardy leguminous shrub to the cool tropical highlands of Africa (Getnet, 1998). It is a perennial browse shrub which is widely grown in Northwest Ethiopia (Getnet, 1998) and has high protein source and it can be an alternative crude protein (CP) supplement for concentrate mixtures. The latter is usually are inaccessible and unaffordable by the majority of smallholder farmers in Ethiopia. Getu et al. (2012) reported that tree lucerne foliage has a 22% CP which is higher than the CP content in wheat bran and lower than the CP content in noug seed cake (*Guizotia abyssinica)*. Because of its CP content tree, lucerne could be included as supplement feed.

Smallholder farmers in the highlands of Ethiopia are currently participating on the Tree lucerne cultivation both in the backyard and natural resource conservation areas for purposes of livestock feed and environmental management (Tadesse, 2016). The utilization of these fodder species as livestock feed, however, is low due to inadequate information particularly about the supplementary value of these feeds. The supplementary potential of a feed resource is chosen based on both in terms of availability and biological response. In the current study biological variables or animal response variables such as nutrient intake, digestibility, growth and carcass characteristics as well as economic parameters were considered due to the fact that these variables are more important for tropical feed evaluation. Moreover, it was difficult to consider other variables because of shortage of facilities and financial limitations. Therefore, the objectives of this study were to evaluate tree lucerne (*Chamaecytisus palmensis*) dried leaves as a substitution for concentrate mixture (wheat bran and noug seed cake) supplement on intake, digestibility, growth and carcass characteristics of Washera sheep fed on desho grass hay as a basal diet.

## 2 MATERIALS AND METHODS

### 2.1 Experimental feeds preparation

Desho grass (*Pennisetum pedicellatum*) was harvested and dried. The grass hay was manually chopped to the size of approximately 5–10 cm in order to facilitate feed intake and minimize wastage then stored under shade to maintain its quality. The sheep were offered ad libitum at 20% refusal rate adjusted every week and provided with free access to clean water throughout the experimental period. Tree lucerne (*Chamaecytisus palmensis*) leaves were harvested from the plantation grown in natural resource conservation area and dried under the shade (air dried). The supplement ratio was made based on their crude protein contribution for growing lamb weight 10–20 kg CP needs range from 16.3% to 26.4% per day recommended by Ensminger (2002). Noug seed cake (NSC) and wheat bran (WB) were purchased from Bahir Dar city oil making factory and floor industry respectively. Diet formulation from these feed ingredients such as TLDL, NSC and WB was done based on the iso‐nitrogenous basis in crude protein content (Ranjhan, 2001). The NSC and WB concentrate mixture was 1:2 ratio.

### 2.2 Experimental sheep and their management

Twenty‐five yearling male Washera sheep with similar initial body weight 16 ± 4.01 kg (mean ± *SD*) were purchased from local market. The experimental animals were ear tagged for identification and quarantined for 15 days in well‐ventilated sheep barn equipped with feeding and watering troughs. Sheep were vaccinated against sheep pox, anthrax and ovine pasteurellosis and injected with multivitamin before commencement of actual experimental period.

### 2.3 Experimental design and treatments

The experiment was conducted in a randomized complete block design (RCBD) with five treatments and five replications. The sheep were divided into five blocks based on their initial body weight which was determined by overnight fasting (to avoid the effect of feed consumed on the weight of animals) and randomly assigned to one of five dietary treatments by using lottery method. The dietary treatments used in the study were presented in Table 1.

**TABLE 1:** Experimental feeds used in the digestibility and growth trials

Treatments	Desho grass hay	Tree lucerne (%)	CM (%)
T_1_	ad libitum	0	100
T_2_	ad libitum	25	75
T_3_	ad libitum	50	50
T_4_	ad libitum	75	25
T_5_	ad libitum	100	0

Abbreviations: CM, concentrate mixture (noug seed cake & wheat bran); TLDL, tree lucerne dried leaves.

### 2.4 Digestibility trial

Digestibility trial was conducted before growth trial. Each sheep were fitted with faecal collection bags for 3 days of acclimatization period followed by a total collection of faeces for 7 consecutive days. Fresh faeces were collected and recorded daily in the morning throughout the digestibility trial. From daily faecal output of each sheep, 20% of the representative samples were taken and frozen at −20ºC, and pooled over the collection period for each sheep.

At the end of the collection period, each sample was taken out from the freeze and allowed to thaw. After thawed, the sample was thoroughly mixed sub‐sample taken and dried at 60ºC for 72 hr. Then grounded into 1 mm sieve size and stored in plastic bags and taken to laboratory for chemical analysis. The apparent digestibility coefficients of different nutrients were calculated by using the following equation:

$$\text{Apparent nutrient digestibility coefficient}\left(\%\right)=\frac{\text{Nutrient intake}‐\text{Faecal nutrient output}}{\text{Nutrient intake}}\times 100,$$

$$\text{Apparent DM digestibility coefficient}\left(\%\right)=\frac{\text{DMI}‐\text{Faecal DM output}}{\text{DMI}}\times 100,$$

Where: DM = dry matter; DMI = dry matter intake.

### 2.5 Growth trial

The 90 days of growth trial was conducted next to digestibility trial. Feed offered and refusals for each sheep were collected and recorded throughout the experimental period to determine feed intake. Daily feed intake was measured as differences between offered and refused. The sheep were offered ad libitum desho grass hay, at 20% (Hozza et al., 2014; Tekliye et al., 2018) refusal throughout the experimental period. Samples of feed offered were collected per batch, whereas samples of refusal were taken from each sheep daily and pooled per treatment over the experimental period and stored in plastic bags used for chemical analysis.

### 2.6 Body weight change and feed conversion efficiency

Body weights were taken every 10 days after overnight fasting before feed provision. Average daily body weight gains (ADBWG) was calculated as the difference between final and initial live weight divided by the number of feeding days. According to Brown et al. (2001), the feed conversion efficiency (FCE) was calculated as unit of body weight gain per unit of feed consumed by the formula;

$$\text{FCE}=\frac{\text{Daily body weight gain}\left(g\right)}{\text{Daily feed intake}\left(g\right)}.$$

### 2.7 Carcass parameters evaluation

At the end of the growth trial, all experimental sheep were slaughtered after overnight fasting to determine the effects of treatment feeds on carcass parameters. Prior to slaughter, body weight was measured to determine the slaughter body weight of each sheep. Empty body weight (EBW) was calculated as the difference between slaughter weight and gut content.

The hot carcass weight (HCW) was estimated after removing the weight of the head, thorax, abdominal, skin and pelvic cavity contents. Dressing percentage was calculated as the proportion of hot carcass weight to slaughter body weight. To evaluate the rib‐eye area, the average of the right and the left side measurement at the 11th and the 12th rib was taken. Total edible offal components (TEOC) were calculated as the total sum of the edible offal components. Total non‐edible offal components (TNEOC) were calculated as the total sum of the non‐edible offal components. All procedures followed were in accordance with the ethical standards of the responsible committee on animal experimentation.

### 2.8 Chemical composition analysis

The feeds offered, refused and faeces samples of the experiment were analysed for DM, OM, CP and Ash contents following the procedures of AOAC (Association of Official Analytical Chemists, 2005). The neutral detergent fibre (NDF), acid detergent fibre (ADF), acid detergent lignin (ADL) and faeces were analysed according to the procedures of Van Soest and Robertson (1985). The metabolizable energy (ME) intake of experimental sheep was estimated from digestible organic matter intake using the formula, ME (MJ/kg) = 0.0157 * DOMI g/kg (AFRC [Agricultural Food & Research Council], 1993), where DOMI = digestible organic matter intake per kg of DM.

### 2.9 Statistical analysis

The data collected on feed intake, digestibility, body weight and carcass were subjected to analysis of variance (ANOVA) using the General Linear Model (GLM) procedure of SAS 2002 (version 9). Analysis of variance among the treatment means, Duncan's Multiple Range Test (DMRT) was used to test and locate the treatment means that significantly differ from the rest. The following model was employed for the experiment:

Model.

$$Y_{\text{ij}}=\mu +t_{i}+b_{j}+e_{\text{ij}},$$

Where Y_ij_ = response variable (an observation in i treatment and j block).

µ = the overall mean.

t_i_ = treatment effect (T1–T5).

b_j_ = block effect (initial body weight effect).

e_ij_ = random error.

### 2.10 Partial budget analysis

The partial budget analysis was performed using the procedure of Upton (1979). It involved the variable cost of purchasing sheep, feeds and selling price of sheep. Total return (TR) was determined by subtracting the selling and purchasing price of sheep. The net income (NI) was calculated as subtracting total variable cost (TVC) from the total return (TR).

NI=TR‐TVC.

**FIGURE 1:**
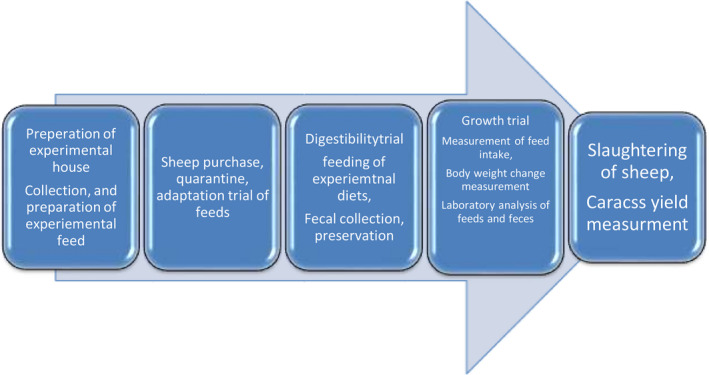
Flow chart of the study

The change in net income (∆NI) was calculated as the difference between changes in total return (∆TR) and the change in total variable costs (∆TVC) and calculated as ∆NI = ∆TR−∆TVC. The marginal rate of return (MRR) that measures the increase in net income (∆NI) associated with each additional unit of expenditure (∆TVC) was calculated as MRR = (∆NI/∆TVC). The flow chart of current study is shown in Figure 1.

## 3 RESULTS

### 3.1 Chemical composition of experimental feeds

The chemical composition of experimental feed and refusals are presented in Table 2. In the current study, the CP content of offered desho grass hay and tree lucerne dried leaves (TLDL) were 11.40 and 23.96%, respectively. As expected, higher fibres were recorded in desho grass hay than other feed ingredients. Similarly, the leftover of grass hay was higher in fibres than the offered, but less in crude protein content.

**TABLE 2:** The chemical composition of experimental feeds and refusals used in the study

Parameters	DM	OM	CP	NDF	ADF	ADL
Feeds						
Desho grass hay (%)	90.0	86.3	11.4	58.6	48.4	14.3
LDL (%)	91.0	95.6	24.1	58.7	35.4	8.6
NSC (%)	91.5	90.5	31.0	34.4	28.5	14.4
WB (%)	91.7	92.3	16.0	39.0	14.4	6.7
CM (%)	91.6	91.4	23.5	36.7	21.4	10.6
Desho grass hay refusal						
T1	90.0	87.8	6.7	60.0	55.9	15.6
T2	96.0	88.5	5.7	63.7	58.9	16.6
T3	96.0	87.5	6.5	65.8	59.0	17.3
T4	96.0	87.5	5.9	68.7	59.9	17.6
T5	95.0	87.4	5.4	70.2	66.8	18.6

Abbreviations: ADF, acid detergent fibre; ADL, acid detergent lignin; CM, concentrate mixture; CP, crude protein; DM, dry matter; NDF, neutral detergent fibre; NSC, Noug seed cake; OM, organic matter; TLDL, tree lucerne dried leaves; WB, wheat bran.

### 3.2 Dry matter and nutrients intake

**FIGURE 2:**
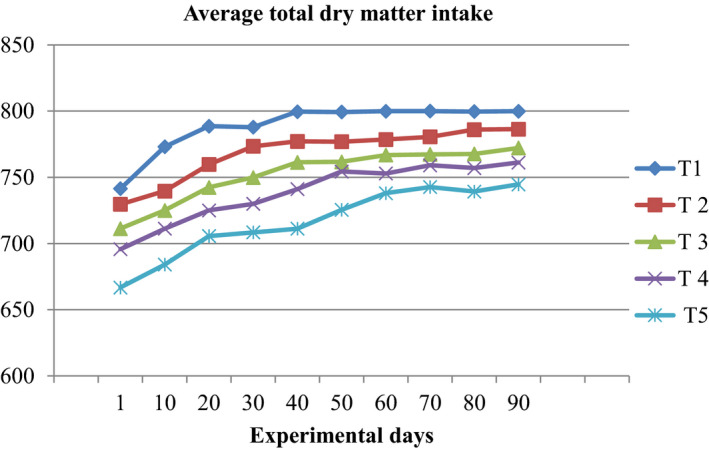
Trends in dry matter (DM) intake over the experimental days for washera sheep fed desho grass hay as a basal diet and supplemented with tree lucerne dried leaves (TLDL) and concentrate mixtures (CM) at different proportions. T1 = 0% TLDL + 100% CM; T2 = 25% TLDL + 75% CM; T3 = 50% TLDL + 50% CM; T4 = 75% TLDL + 25% CM; T5 = 100% + 0% CM. T1–T5 = Treatment

Significant (*p* < .05) difference were observed among treatments except DMI% body weight, total NDFI and total ADFI of the daily nutrients intake (Table 3); as the level of TLDL increase in the diet of the TDM, ME, TOM and TCP intake were in decreasing order. The highest nutrient intake (813 g/day) was recorded in sheep assigned in T1 and lowest (740 g/day) in T5 (sole TLDL supplement). In the current study, there was no significant (*p* > .05) differences in nutrient intake among the TLDL‐supplemented groups. All treatments group had similar (*p* > .05) NDF and ADF intake, but high level (103 g/day) of lignin consumed in the sole TLDL assigned sheep. The ADL intake significantly (*p* < .05) increased as the level of TLDL increased; Sheep fed sole TLDL had higher consumption than the groups fed non‐TLDL diet. A trend of total DM intake is shown in Figure 2.

**TABLE 3:** Daily dry matter and nutrients intake of Washera sheep fed Desho grass hay as a basal diet and supplemented with tree lucerne dried leaves and concentrate mixtures at different proportion

Parameters	Treatments							
Intake(g/day)	T1	T2	T3	T4	T5	*SEM*		SL
Basal DMI (g/day)	393.4^a^	357.8^ab^	340.1^ab^	331.3^ab^	320.0^b^	3.5		*
TLDL DMI (g/day)	‐	98.0^d^	196.0^c^	294.0^b^	392.0^a^	0.0		***
CM DMI (g/day)	420.0^a^	315.0^b^	211.0^c^	105.0^d^	‐	0.0		***
Total DMI (g/day)	813.4^a^	777.8^ab^	760.1^ab^	751.3^ab^	740.0^b^	11.5		*
DM intake (%BW)	3.4	3.4	3.5	3.6	3.6	0.0		NS
Nutrient intake								
ME (MJ/d)	7.3^a^	6.4^b^	6.0^bc^	5.8^bc^	5.2^c^	0.2		*
TOMI (g/day)	760.5^a^	727.4^ab^	694.6^ab^	673.8^b^	666.3^b^	9.7		*
TCPI (g/day)	118.3^a^	113.6^ab^	109.3^ab^	105.6^bc^	98.6^c^	3.0		**
TNDFI (g/day)	342.2	345.7	345.4	340.9	356.6	6.3		NS
TADFI (g/day)	257.1	259.0	262.2	268.9	276.3	5.1		NS
TADLI (g/day)	86.3^b^	88.0^b^	89.3^b^	96.6^ab^	103.4^a^	3.4	*	

Note

^a,b,c,d^Means with different superscripts in a row are significantly different at *=(*p* < .05); ***=(*p* < .001); non‐significant.

Abbreviations: ADFI, acid detergent fibre intake; ADLI, acid detergent lignin intake; CM, concentrate mixture; CPI, crude protein intake; DMI, dry matter intake; ME, metabolizable energy; MJ/d, mega joule per day; NDFI, neutral detergent fibre intake; OMI, organic matter intake; *SEM*, standard error of mean; SL, significance level; TLDL, tree lucerne dried leaves.

### 3.3 Apparent dry matter and nutrient digestibility

The apparent dry matter and nutrients digestibility percentage of experimental feeds are shown in Table 4. Like the daily dry matter and nutrients intake, digestibility percentage of dry matter and OM and CP nutrients significantly (*p* < .05) decreased as the level of TLDL increased in the diet. For these nutrients, the higher nutrients digestibility was for T1 and lowest for T5. Non‐significant (*p* > .05) values were observed for NDF and ADF values.

**TABLE 4:** Apparent dry matter and nutrient digestibility of Washera sheep Desho grass hay as a basal diet and supplemented with tree lucerne dried leaves and concentrate mixtures at different proportion

Digestibility (%)	Treatments					*SEM*	SL
	T1	T2	T3	T4	T5		
DM	59.5^a^	55.4^ab^	54.0^bc^	53.8^bc^	49.7^c^	0.9	*
OM	60.5^a^	55.0^ab^	54.0^b^	53.7^b^	48.7^c^	1.0	**
CP	75.6^a^	72.4^a^	69.3^a^	62.3^b^	58.9^b^	1.5	**
NDF	50.1	44.4	43.2	42.1	41.3	1.7	NS
ADF	46.2	42.6	41.4	40.1	39.9	1.5	NS

Note

^a,b,c^Means in the same row with different superscripts are different at *=*p* < .05; **=*p* < .01.

Abbreviations: ADF, acid detergent fibre; CM, concentrate mixture; CP, crude protein; DM, dry matter; NDF, neutral detergent fibre; OM, organic matter; *SEM*, standard error of mean; SL, significance level; T = treatment TLDL; tree lucerne dried leaves.

### 3.4 Body weight and feed conversion efficiency

**FIGURE 3:**
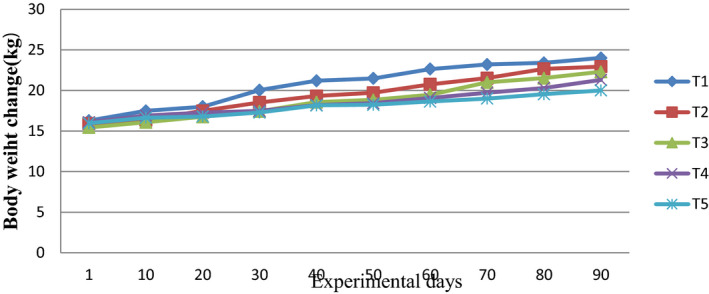
Trends in BW(body weight) changes across the experimental days for Washera sheep fed desho grass hay and Supplemented with tree lucerne dried leaves (TLDL) and CM (concentrate mixture) in different proportions.T1 = 0% TLDL + 100% CM; T2 = 25% TLDL + 75% CM; T3 = 50% TLDL + 50% CM; T4 = 75% TLDL + 25% CM; T5 = 100% TLDL + 0% CM. T1–T5 = Treatment

Significant (*p* < .05) on body weight change (BWG), average daily gain (ADG) and feed conversion efficiency (FCE) were recorded and presented in Table 5. Sheep fed on the lower level of TLDL (T1–T2) included diet had better BWG (6.3–7.4 kg), ADG (71–82 g/day) and FCE (0.09–0.1) than the sole TLDL diet (T5) which has lowest record 4.6 kg, 51 g/day and 0.07 for BWG, ADG and FCE respectively. On the other side, however, there was no significant (*p* > .05) difference among the TLDL‐treated diets (T2, T3 and T4) in terms of sheep growth performances. The trends of body weight change in experimental sheep are shown in Figure 3.

**TABLE 5:** Body weight change and feed conversion efficiency of Washera sheep fed Desho grass hay as a basal diet and supplemented with tree lucerne dried leaves and concentrate mixtures at different proportion

Parameters	Treatments					*SEM*	SL
	T1	T2	T3	T4	T5		
Initial body weight (kg)	16.4	16.3	16.3	16.2	16.2	0.2	NS
Final body weight (kg)	23.8	22.6	21.5	21.2	20.8	0.3	NS
Body weight change (kg)	7.4^a^	6.3^ab^	5.2^bc^	5.0^bc^	4.6^c^	0.3	*
ADG (g/day)	82.2^a^	71.1^ab^	57.7^bc^	55.5^bc^	51.1^c^	2.3	*
FCE (g ADG/g TDMI)	0.1^a^	0.1^ab^	0.1^bc^	0.1^bc^	0.07^c^	0.0	*

Note

^a,b,c^Means in the same row with different superscripts are different at *=*p* < .05; NS = non‐significant.

Abbreviations: ADG, average daily gain; CM, concentrate mixture; FCE, feed conversion efficiency; *SEM*, standard error of means; SL, significance level; TDMI, total dry matter intake; TLDL, tree lucerne dried leaves.

### 3.5 Correlation among nutrients intake, digestibility and daily weight gain of Washera sheep

Dry matter intake was positively and strongly correlated (*p* < .0001) with OM intake as well as OM digestibility (Table 6). DMI was also positively moderately correlated (*p* < .01) with CP intake and DM, CP and NDF digestibility. The CP intake was positively moderately correlated (*p* < .05) with intake of DM and OM, digestibility of CP (*p* < .05) and positively weakly correlated with NDF and ADF digestibility. Crude protein digestibility was positively (*p* < .05) correlated moderately with DM, OM weakly and CP intake. The CP digestibility also positively moderately correlated with DM and OM digestibility, whereas it is positively weakly correlated with ADFD (*p* < .05). ADG showed positive strong correlation with OM, CP and NDF digestibility values and positively moderately correlated with DM intake and digestibility, OM digestibility and CP intakes. However, ADG had positive very weak correlation with NDF and ADF intake and ADF digestibility values.

**TABLE 6:** Pearson correlation between intake, digestibility and body weight gains of Washera sheep fed Desho grass hay as a basal diet and supplemented with tree lucerne dried leaves and concentrate mixtures at different proportion

Parameters	DMI	DMD	OMI	OMD	CPI	CPD	NDFI	NDFD	ADFI	ADFD	ADG
DMI	1.00	0.40^*^	0.66^***^	0.67^***^	0.47^*^	0.47^*^	0.22^NS^	0.47^*^	0.22^NS^	0.56^**^	0.53^**^
DMD		1.00	0.58^**^	0.63^***^	0.48^*^	0.49^*^	−0.11	0.22^NS^	−0.12	0.36^*^	0.41^*^
OMI			1.00	0.70^***^	0.42^*^	0.46^*^	0.10^NS^	0.41^*^	0.14^NS^	0.39^*^	0.48^*^
OMD				1.00	0.43^*^	0.59^**^	0.08^NS^	0.50^*^	0.05^NS^	0.43^*^	0.61^**^
CPI					1.00	0.35^*^	−0.14	0.15^NS^	−0.14	0.12^NS^	0.43^*^
CPD						1.00	−0.31	0.30^NS^	−0.37	0.34^*^	0.61^**^
NDFI							1.00	0.65^***^	0.95^***^	0.29^NS^	0.06^NS^
NDFD								1.00	0.52^**^	0.47^*^	0.61^**^
ADFI									1.00	0.31^NS^	0.06^NS^
ADFD										1.00	0.13^NS^
ADG											1.00

Abbreviations: ADFD, acid detergent fibre digestibility; ADFI, acid detergent fibre intake; ADG, average daily weight gain; CPD, crude protein digestibility; CPI, crude protein intake; DMD, dry matter digestibility; DMI, dry matter intake; NDFD, neutral detergent fibre digestibility; NDFI, neutral detergent fibre intake; NS, non‐significant; OMD, organic matter digestibility; OMI, organic matter intake.

* =*p* < .05;

** =*p* < .01;

*** =*p* < .001.

### 3.6 Carcass characteristics

Non‐significant (*p* > .05) differences were observed among treatments in most of the carcass characteristics of Washera sheep fed desho grass hay basal diet supplemented with different levels of TLDL and CM mixture except for rib‐eye area (Table 7). Sheep fed on T1 and T2 relatively had better (12 cm^2^) rib‐eye area (REA) than the sole TLDL‐supplemented group (T5). Non‐significant (*p* > .05) difference recorded among the groups supplemented with higher levels of TLDL which are in the T3, T4 and T5. (Table 8). In the current study, rib eye muscle and ribs were significant (p < .05) with the higher value for lower TDL supplemented groups (Table 8).

**TABLE 7:** Carcass characteristics of Washera sheep fed Desho grass hay as a basal diet and supplemented with tree lucerne dried leaves and concentrate mixtures at different proportion

Parameters	Treatments					*SEM*	SL
	T1	T2	T3	T4	T5		
SW (kg)	24.1	22.6	21.6	21.3	21.0	0.4	NS
EBW (kg)	19.8	18.5	17.5	17.5	17.3	0.4	NS
HCW (kg)	9.3	8.9	8.7	8.6	8.5	0.2	NS
Dressing percentage:							
SW basis (%)	38.4	39.5	40.2	40.4	40.8	0.5	NS
EBW basis (%)	47.0	48.0	50.0	49.0	49.0	0.2	NS
REA^a^ (cm^2^)	12.6^a^	12.2^a^	11.0^b^	10.4^bc^	10.1^bc^	0.3	**

Note

^a,b,c^ = means within a rows having different superscript are significantly different at **=*p* < .05.

Abbreviations: CM, concentrate mixture; EBW, empty body weight; HCW, hot carcass weight; REA, rib eye area; *SEM*, standard error of mean; SL, significant level; SW, slaughter weight; TLDL, tree lucerne dried leaves.

**TABLE 8:** The main carcass component Washera sheep fed Desho grass hay as basal diet and supplemented with tree lucerne dried leaves and concentrate mixtures at different proportion

Main carcass components	Treatments					*SEM*	SL
	T1	T2	T3	T4	T5		
Fore quarter (g)	2,051.7	1,949.3	1,845.0	1,806.7	1,764.7	7.6	NS
Neck region (g)	462.4	460.0	450.1	449.5	446.2	4.9	NS
Sternum (g)	273.6	271.3	253.6	241.0	234.3	1.6	NS
Thoracic and lumbar (g)	574.3	572.3	492.6	490.3	482.6	9.6	NS
Rib eye muscle (g)	659.4^a^	615.2^ab^	580.7^ab^	555.3^b^	506.2^c^	15.9	*
Abdominal muscle (g)	466.6	429.0	422.3	420.3	419.1	12.7	NS
Hind quarter (g)	2,420.3	2,198.0	2,179.3	2,105.0	2,094.7	24.2	NS
Pelvic region (g)	573.3	529.3	526.0	515.0	503.3	4.5	NS
Ribs (g)	530.6^a^	498.0^ab^	460.0^bc^	457.0^bc^	431.0^c^	2.5	**
Tail fat (g)	1,100.1	1,031.3	956	926.7	840.4	29.4	NS
TMCC (g)	8,122.6	7,625.7	7,305.5	7,132.9	6,982.5	16.5	NS

Note

Means within rows having different superscript letters are significantly different *(*p* < .05); **=*p* < .01.

Abbreviations: CM, concentrate mixture; NS, not significant; *SEM*, standard error of mean; SL, significant level; TLDL, tree lucerne dried leaves; TMCC, total main carcass components.

#### 3.6.1 Edible offal components

Among the edible offal components, significant effect of diet (*p* < .05) was observed on liver, kidney, tongue, omaso‐abomasum and omental mesenteric fat, whereas for other offal components there was no significant (*p* > .05) difference among the treatments (Table 9). Like other carcass characteristics, sheep assigned in T1 had higher liver (25.92%), kidney (41.76%), tongue (11.89%), omaso‐abomasum (32.03%) and omental mesenteric fat (110.79%) higher than for the values of sheep in T5.

**TABLE 9:** The edible offal component of Washera sheep fed Desho grass hay as a basal diet and supplemented with tree lucerne dried leaves and concentrate mixtures at different proportions

Edible offal	Treatments					*SEM*	SL
	T1	T2	T3	T4	T5		
Blood (g)	879.0	875.3	868.3	866.2	832.6	21.9	NS
Heart (g)	94.7	92.4	87.6	85.7	83.8	2.0	NS
Liver (g)	352.7^a^	348.5^ab^	297.2^b^	293.03^b^	280.1^c^	5.7	*
Kidney (g)	64.5^a^	60.1^ab^	59.4^ab^	48.9^bc^	45.5^bc^	1.1	*
Tongue (g)	100.7^a^	110.5^a^	91.8^ab^	91.7^ab^	90.0^b^	2.3	*
Reticulo‐rumen (g)	529.8	485.5	481.0	480.9	478.3	4.6.	NS
Omaso‐abomasum (g)	185.1^a^	177.4^a^	158.5^ab^	141.3^b^	140.2^b^	6.4	*
Hind gut (g)	811.1	788.5	752.8	651.3	627.7	5.8	NS
Testicles (g)	263.2	243.5	221.1	217.1	205.2	6.8	NS
Kidney fat (g)	34.8	31.0	26.0	25.0	21.0	2.7	NS
Omental mesenteric fat (g)	72.3^a^	43.0^b^	41.0^b^	36.0^bc^	34.3^bc^	5.0	*
Total edible offal (kg)	3.3	3.1	3.0	2.9	2.8	0.1	NS
Total usable product (kg)	13.9	13.2	12.6	12.3	11.9	0.3	NS

Note

^a,b,c^ = Means within rows having different superscript letters are significantly different *(*p* < .05).

Abbreviations: CM, concentrate mixture; NS, non‐significant; *SEM*, standard error of mean; SL, significance level; TLDL, tree lucerne dried leaves.

#### 3.6.2 Non‐edible offal components

The non‐edible offal components of *Washera* sheep fed desho grass hay as a basal diet and supplemented with tree lucerne dried leaves and concentrate mixtures at different proportion are presented in Table 10. Some non‐edible offal component was not significantly different (*p* > .05). But gull bladder with bile and bladder were significantly different (*p* < .05). Highest bladder (43 g) and gut content (4,275 g) recorded in sheep assigned in T1 and lowest in the T5 ones.

**TABLE 10:** Non‐edible offals of Washera sheep fed Desho grass hay as a basal diet and supplemented with tree lucerne dried leaves and concentrate mixtures at different proportions

Non‐edible offal	Treatments					*SEM*	SL
	T1	T2	T3	T4	T5		
Head without tongue (g)	1,167.6	1,128.0	1,118.3	1,103.6	1,063.6	23.5	NS
Skin (kg)	2.5	2.4	2.3	2.2	2.1	0.1	NS
Penis (g)	63.5	60.3	56.4	49.8	48.9	2.9	NS
Feet (g)	540.1	527.5	504.0	501.6	488.0	9.3	NS
Lung with trachea (g)	306.8	297.6	283.6	283.4	266.7	6.4	NS
Spleen (g)	43.2	35.3	33.5	32.0	30.4	2.0	NS
Oesophagus (g)	39.9	35.4	30.2	30.1	29.8	2.5	NS
Gull bladder with bile (g)	28.4	23.9	19.5	17.1	15.6	2.2	NS
Bladder	43.5^a^	40.7^ab^	34.9^abc^	28.6^bc^	24.1^c^	2.5	*
Gut content (g)	4,275.7^a^	4,123.8^ab^	4117^ab^	3,847.1^bc^	3,732.0^bc^	26.9	*
Pancreas (g)	37.0	31.5	31.0	30.0	29.4	1.7	NS
Total non‐edible offal (kg)	6.5	6.2	6.2	6.1	5.9	0.1	NS

Note

^a,b,c^ = Means within rows having different superscript letters are significantly different *(*p* < .05).

Abbreviations: CM, concentrate mixture; NS, non‐significant; *SEM*, standard error of mean; SL, significance level; TLDL, tree lucerne dried leaves.

### 3.7 Partial budget analysis

The partial budget analysis results of Washera sheep fed desho grass hay as a basal diet and supplemented with TLDL and concentrate mixtures in different proportion are presented in Table 11. The total return per animal of treatment effect was lower than concentrate mixtures, where it was better for the concentrate mix supplement treatment group (T1) followed by mixed supplement and TLDL alone respectively. The net returns from the supplemented treatments for T1, T2, T3, T4 and T5 were 31.66, 30.68, 30.34, 27.79 and 26.32 USD/head respectively. The marginal rate of return (MRR) was 0.062, 0.060, 00.092 and 0.0.097 USD for T2, T3, T4 and T5 respectively.

**TABLE 11:** Partial budget analysis

Variables	Treatment				
	T1	T2	T3	T4	T5
Number of animals	5.00	5.00	5.00	5.00	5.00
Purchase price of sheep (USD/head)	32.25	32.25	32.18	32.18	32.11
Total feed consumed (kg/head)	200.60	164.90	125.40	97.20	64.00
Total basal diet consumed (kg/head)	35.40	32.40	30.60	29.80	28.80
Total TLDL consumed (kg/head)	0.00	8.80	17.60	26.50	35.30
Total CM consumed (kg/head)	165.20	123.90	77.00	40.90	0.00
Cost of basal diet (hay) (USD/head)	35.40	32.20	30.60	29.80	28.80
Cost of TLDL (USD/head)	0.00	35.20	70.50	105.80	141.00
Cost of CM (USD/head)	6.72	5.04	3.27	1.67	0.00
Total feed cost (USD/head)	7.98	7.44	6.86	6.49	6.04
Gross income (selling price of sheep) (USD)	71.92	70.43	69.43	66.51	64.52
Total return (USD)	39.62	38.13	37.20	34.29	32.37
Net return (USD)	31.66	30.68	30.34	27.78	26.32
ΔTVC	‐	−0.54	−1.12	−1.49	−1.94
ΔNI	‐	−0.96	−1.32	−3.88	−5.34
MRR (ΔNI/ΔTVC)	‐	0.06	0.04	0.09	0.10

Abbreviations: MRR, marginal rate of return; USD, United States Dollar; ΔNI, change in net income; ΔTVC, change in total variable cost.

## 4 DISCUSSION

### 4.1 Chemical composition of experimental feeds

In the current study, the CP content of desho grass hay is relatively higher than the value of the previous result reported by Asmare et al. (2016) 8.6%, 7.5% planted in the mid and high altitude areas of Ethiopia respectively. The deviation of current result from previous reports might be due to drying process, storage, stage of maturity and differences in the growing environment (Biniyam et al., 2018). The CP content of tree lucerne dried leaves was in the range of values (21.6%–27.8%) as reported by Dereje (2014) but higher than (16.6%) the reports of Meron (2016). The NDF, ADF and ADL content of Tree Lucerne leaves were in line with the previous study (Getu et al., 2012). The DM, OM, CP, NDF, ADF and ADL content of the Desho grass hay refusals were almost similar among treatments. This might be due to the feeding habits of sheep which selected the most nutritious parts of the grass such as leaves, shoots and left relatively less edible parts like stems of the grass (Alemu, 2016).

The CP content of noug seed cake (NSC) in the current study was similar to the range of values (30%–32%) noted by different authors (Aniteheh et al., 2015; Taye, 2011). The NDF content of NSC (34.4%) was higher than 28.3% reported by Kefyalew et al. (2015), whereas the ADF content (28.5%) was relatively comparable with the value (30.1%) documented by Amade (2015). The ADL content (14.4%) was higher than 6.7% noted by Worknesh and Getachew (2018) for different grass species. The CP content of wheat bran (16%) was aligned to the value of 17.40% and 17.20% reported by different authors (Kefyalew et al., 2015; Worknesh & Getachew, 2018). The difference in chemical composition of NSC and WB of the current finding from previous results might be due to variation in the processing methods of extraction and milling, quality of the raw material and growing condition of the forage crop (Solomon, 1992).

### 4.2 Dry matter and nutrients intake

The total DM, OM, CP and ADL intake result of sheep in the current study were in agreement with the result of Gebregiorgis et al. (2017) fed *Leucaena leucocephala* leaves and pods and wheat bran for central‐highland sheep in Ethiopia.

The mean total DM intake was comparable with the range of values from 768 to 979 g/day reported by Hirut et al. (2011) for Hararghe highland sheep fed urea‐treated maize straw with different level of concentrate mixture. The current finding, however, is higher than the range of values from 581.7 to 729.9 g/day reported by Awoke (2015) for the same breed of sheep fed hay as a basal diet and supplemented with *F.sycomorous* leaf, fruit and their mixtures. Generally, the total DM intake (% body weight) was within the range of 2%–6% recommended for sheep (ARC [Agricultural Research Council], 1980).

The metabolizable energy (ME) requirement for a 10 kg live weight sheep has been documented to be 2 MJ/day for maintenance and 2.7–4.5 MJ/day ME for 50–150 g/day gain (Chesworth, 1992). In the current study, ME in the feed was in line with the finding of Mekuriaw and Asmare, (2018) who reported values ranging from 7.12 to 7.48 MJ/day for the same breed of sheep. The estimated ME intake of sheep in the current study might be associated with organic matter in desho grass hay and other supplemented feeds. The CP intake in the current study ranges from 118.3 to 98.6 g/day which was lower than the recommendation of 124 g CP/kg DM for a 20 kg growing lamb at 200 g/head/day weight gain (ARC, 1980) but, the CP intake is in the range reported by Getahun (2015) for fattening yearling Arsi‐Bale lambs. The discrepancy of current finding from the previous results might be due to the fact that the feed in this study contained relatively low CP and high fibre content. However, the current result was analogous to the result of Alemayehu et al. (2015) and Feleke et al. (2011) who reported the CP intake was in the range of values from 54.23 to 111.98 and 17.8 to 130.8 g/day supplemented with different by‐products and with different sheep breeds. The NDF and ADF intakes of sheep were comparable with the result of Mekuriaw and Asmare (2018) which was within the range 222.56–278.89 g/day for the same breed of sheep.

### 4.3 Apparent dry matter nutrient digestibility

Regarding digestibility of nutrients, there was lower digestibility of DM, OM and CP in sheep fed only tree lucerne dried leaves compared with all the treatments which might be due to relatively lower energy and higher fibre contents of the feed. It has been stated that there would be variation in DM and OM apparent digestibility nutrients in a given diet that arise from variation related to the age of the animals, level of feeding, feed nutrient content and ration composition (Ranjhan, 2001).

In the current finding, the DM digestibility was in line with the value which ranges from 0.67% to 0.52% (Dereje, 2014) in the evaluation of Tree Lucerne forages as a substitute for concentrate in diets of local sheep fed natural pasture hay. On the other hand, the current result was relatively lower than the result of Awoke (2015) fed natural pasture hay supplemented with *Ficus sycomorus* for same breed of sheep. The CP digestibility of sheep fed mixed supplement, and tree lucerne group is relatively comparable with the value (67.9%) reported by Hagos (2015) for Tigray local sheep fed on hay and supplemented with *Sesbania sesban* leaves. The current finding, however, is higher than the values reported by Teklu, Adunga, Jane, Getachew, & Barbara, (2018) that ranges from 47.5 to 61.9 g/day for Arsi‐Bale sheep fed different varieties of faba bean (*Vicia faba L*.) straws supplement with concentrate mix. The observed CP digestibility which was higher in (T1) might be due to the lower fibre fraction, and high CP and energy concentration of the diets that in turn improved the digestibility of nutrients. Nevertheless, the digestibility of NDF and ADF in the current finding was in agreement to the result of McRae and Armstrong (1969) who reported that supplementation had little or no effect on digestibility of NDF.

### 4.4 Body weight change and feed conversion efficiency

In the present study, insignificant results for initial and final body weight recorded were similar to the reports of Desta et al. (2017) for Abergele rams in a study conducted to substitute local concentrate mix with dried mulberry (*Morus indica L*.) leaf meal. The body weight change, average daily gain (ADG) and feed conversion efficiency values were in agreement with the findings of Mekonnen et al. (2017) for a research that focused on the effect of Mulberry Leaf meal supplementation for Ethiopian Highland sheep.

The lower body weight of sheep in T5 indicated that tree lucerne dried leaves provided lowest metabolizable energy (5.2 MJ/day) mainly for maintenance requirement of sheep rather than body weight gain. This was supported by Gatenby (2002) who stated that the lowest energy at which the sheep do not lose weight is between 8 and 10 MJ/kg per head/day. In addition, the minimum protein level required for maintenance is about 8% CP in the DM and the most productive animals such as rapidly growing lambs and lactating ewes need about 11% CP (McDonald et al., 2010). The lower weight gain of sheep supplemented with solely tree lucerne leaves could be due to the inefficient digestibility of TLDL associated with the high fibre content than concentrate mix (Dereje, 2014). In the current study, the mean daily body weight gain was relatively comparable with reports of Awet and Solomon (2009) who also reported 65–88.2 g/day in Afar rams supplemented with 150–350 g wheat bran/day. In the current finding, however, the value is higher than the result of Tekliye et al. (2018) with the range of values from 19.33 to 45.11 g/day fed urea‐treated rice straw supplemented with graded levels of dried *Sesbania sesban* leaves for Farta sheep. The FCE is in line with earlier study (Gebregiorgis et al., 2017) (0.08–011) for central‐highland sheep fed *Luciana leucocephala* leaves and pods and wheat bran.

### 4.5 Correlation among nutrients intake, digestibility and daily body weight gain of Washera sheep

Sheep fed on higher protein diet consumed more total DM than sheep kept on low CP diet (Fluharty et al., 1999). This is in agreement with the results reported by Aschalew and Getachew (2013) for Farta sheep. The positive associations between total DM and CP intakes were also reported by Awet and Solomon (2009) for Afar sheep. The NDF and ADF intake were negatively and non‐significantly correlated with the digestibility of DM, OM and CP. This implies that a high intake of fibre fractions reduced the digestibility of DM, OM as well as CP, which in turn reduces intake of feed (Martens, 2006).

### 4.6 Carcass parameters

The relatively higher SW, EBW and HCW in T1 than other treatments might be due to the fact that concentrate mix can easily be digestible and higher nutrients consumption by sheep in T1 which was consistent with ADG and feed conversion efficiency of sheep in the treatment. Similar finding was reported by Desta et al. (2017) for Abergele rams supplemented with substitution of dried mulberry (*Morus indica* L.) leaf meal for concentrate mix. In the present study, the SW values were relatively heavier than the results of Dereje (2014) and Tekliye et al. (2018) 17.2–20.6 and 17.52–22.60 kg for local and Farta sheep, respectively, supplemented with Tree Lucerne Sesbania sesban fodder leaves. Hot carcass weight was relatively comparable with reported values of 5.6–9.6 and 9.75–11 kg for Arsi‐Bale and local sheep reported by Abebe and Yoseph (2015) and Hagos (2015) respectively. Furthermore, EBW of the current finding is similar to the result of Awoke. (2015) who reported a range of values from 14.4 to 17.75 kg supplemented with Ficus sycomorus leaves for Washera sheep. On the contrary, however, the current result was higher than the values reported by Berhanu et al. (2014) with the range from 11.9 to 12.3 kg supplemented with Millettia ferruginea (Birbra) foliage for Washera sheep.

In the current study, dressing percentage on slaughter weight basis records of treatments was not significantly different (*p* > .05) which was similar to the finding of Assefu (2012) for Washera and Horro sheep fed on roughage and concentrate mix. The value of dressing percentage as a proportion of SW and EBW basis result showed that dressing percentage on EBW basis was higher than on SW basis which illustrates the influence of gut fill (Gibbs & Ivings, 1993) who reported that ingesta constitute a large portion of the body weight even when the animals fast for long hours. The mean value of SW basis was in line with the range value from 32.73 to 48.51 and 38.3% to 43.6% for the reports of Yilkal et al. (2014) and Aschalew and Getachew (2013) supplemented with different forms of processed Lupin (Lupinus albus) grain and raw, malted and heat‐treated grass pea (Lathyrus sativus) for Washera and Farta sheep respectively.

Dressing percentage on EBW basis in this study was higher than the results (38.1%–38.3%) reported by Gebru et al. (2015) fed Rhodes grass (*Chloris gayana*) hay supplemented different forms of white Lupin (*Lupinus albus*) grain for the same breed. The dressing percentage (EBW basis) values of the current study were found within the range of values of the average dressing percentages (ADP) of tropical sheep (40%–50%) reported by (William et al., 2003). The rib‐eye area muscles were consistent with the results of Alemayehu et al. (2015) and Gebregiorgis et al. (2017) fed *Leucaena leucocephala* leaves and pods and wheat bran for Central‐highland sheep. Higher rib‐eye muscles area was observed in T1 12.6 cm^2^ compared with other treatments. This indicates that the nutritional quality of supplementation would enable sheep to develop better muscling than the other group (Amare, 2007). This might be due to the higher nutrients provided by the supplements with CM and higher slaughter weight. The rib‐eye area (REA) was relatively comparable with the result of Girma and Mengistu (2017) in the range value 5.92%–14.7% for Horro sheep fed urea‐treated maize husk and untreated maize husk supplemented with different levels of concentrate mixture.

#### 4.6.1 Main carcass components

Rib and rib‐eye muscles were relatively higher groups in T1 than other treatments. This might be due to sheep in this treatment could have consumed high quality of feed and easily digestible that contain protein and energy (Kouakouet al., 1997) who reported that the weight of some visceral organs is affected by the level of nutrition.

#### 4.6.2 Edible offal components

Liver, Kidney, Tongue and Omaso abomasum were higher in sheep in T1 and lower T4 and T5. This might be due to high metabolic activities of the organs and high intake of energy and protein from concentrate supplemented sheep in T1. The non‐significant differences in most of the alimentary canals could be linked with organs being early maturing and consequently being little affected by dietary treatments (Hag & Shargi, 1996).

Total edible offal components and total usable products were relatively higher in T1 (3.3 kg) sheep fed with concentrate mix only compared with others, even though non‐significant among the treatments. In agreement with previous studies (Abebe et al., 2010; Hailu et al., 2014; Hirut et al., 2011; Meron, 2016) who reported that heavier weight of edible offal components for supplemented groups than the non‐supplemented group. The current study TEOC was (2.8–3.3 kg) relatively comparable with the result of Hirut et al. (2011); (2.22–4.19 kg) supplemented and non‐supplemented sheep respectively.

#### 4.6.3 Non‐edible offal components

Among non‐edible offal components, skin weight of T1 (2.5 kg) was higher than T5 which might be due to the better subcutaneous layer fat deposition due to better feeding regime sheep supplemented with concentrate mixture and better growth of hair (Lawrence & Fowler, 1998).

### 4.7 Partial budget analysis

The net return of supplemented sheep in the current study was relatively higher compared to the results reported by previous workers (Melese et al., 2014; Tesfaye, 2008) which was in the range of 52.00–82.16 and 60.10–153.20 ETB/head for different sheep breeds fed various basal and supplement diets. The observed difference in net return might be due to the variations in purchasing and selling price of sheep. The sheep in T5, however, gained lower BW as a result of lower nutrient (CP) and energy with higher fibre content intake that consequently resulted in a lower net return. The MRR result achieved in the present study was higher than 0.9–1.9 and 1.2–2.0 reported by Melese et al. (2014) and Tesfaye (2008) for Washera and Arsi‐ Bale sheep, respectively, fed different basal diet and supplements. This might be due to variations in sheep breeds and differences in basal diet and supplements used in different experiments. As a limitation of this study, only few common variables were considered in this study due to constraints like laboratory and other inputs.

## 5 CONCLUSION

From the present study, it could be concluded that substitution of tree lucerne dried leaves by conventional concentrate mix (NSC & WB) up to 75% inclusion because it satisfies the nutrient requirement of fattening sheep which were biologically efficient and economically profitable. For commercial producers, however, up to 50% inclusion of TLDL in the diet of growing local sheep could be recommended. Therefore, TLDL could sustainably serve as alternative CP supplements to the roughage feed stuffs as a basal diet for ruminant animals in the smallholder farming systems where concentrate mix is not affordable and accessible.

## STUDY AREA

The study was conducted at the College of Agriculture and Environmental Sciences, Bahir Dar University, Ethiopia. The area is geographically located at 11° 37′ N and 37° 28′ E with an elevation of 1912 meter above sea level. The average daily minimum and maximum temperatures were 7 and 29^◦^C respectively. The average annual rainfall ranges were from 1,430 to 1,520 mm.

## CONFLICT OF INTEREST

The authors declare that they have no conflict of interest.

## AUTHOR CONTRIBUTION

**Shewaye HaileCherkos:** Conceptualization; Data curation; Formal analysis; Methodology; Software. **Bimrew Asmare:** Conceptualization; Methodology; Supervision; Writing‐original draft; Writing‐review & editing. **Yeshambel Mekuriaw:** Conceptualization; Data curation; Methodology; Supervision; Writing‐original draft; Writing‐review & editing.

### PEER REVIEW

The peer review history for this article is available at https://publons.com/publon/10.1002/vms3.376.

## Data Availability

Data are available if it is required for review.
